# Novel SSR Markers from BAC-End Sequences, DArT Arrays and a Comprehensive Genetic Map with 1,291 Marker Loci for Chickpea (*Cicer arietinum* L.)

**DOI:** 10.1371/journal.pone.0027275

**Published:** 2011-11-15

**Authors:** Mahendar Thudi, Abhishek Bohra, Spurthi N. Nayak, Nicy Varghese, Trushar M. Shah, R. Varma Penmetsa, Nepolean Thirunavukkarasu, Srivani Gudipati, Pooran M. Gaur, Pawan L. Kulwal, Hari D. Upadhyaya, Polavarapu B. KaviKishor, Peter Winter, Günter Kahl, Christopher D. Town, Andrzej Kilian, Douglas R. Cook, Rajeev K. Varshney

**Affiliations:** 1 Grain Legumes Research Program, International Crops Research Institute for the Semi-Arid Tropics (ICRISAT), Hyderabad, India; 2 Department of Genetics, Osmania University, Hyderabad, India; 3 Department of Plant Pathology, University of California Davis, Davis, California, United States of America; 4 Division of Genetics, Indian Agricultural Research Institute, New Delhi, India; 5 State Level Biotechnology Centre, Mahatma Phule Agricultural University, Ahmednagar, India; 6 GenXPro GmbH, Frankfurt am Main, Germany; 7 Molecular BioSciences, University of Frankfurt, Frankfurt am Main, Germany; 8 J. Craig Venter Institute (JCVI), Rockville, Maryland, United States of America; 9 DArT Pty. Ltd., Yarralumla, Australia; 10 CGIAR Generation Challenge Programme (GCP), CIMMYT, Mexico DF, Mexico; Lund University, Sweden

## Abstract

Chickpea (*Cicer arietinum* L.) is the third most important cool season food legume, cultivated in arid and semi-arid regions of the world. The goal of this study was to develop novel molecular markers such as microsatellite or simple sequence repeat (SSR) markers from bacterial artificial chromosome (BAC)-end sequences (BESs) and diversity arrays technology (DArT) markers, and to construct a high-density genetic map based on recombinant inbred line (RIL) population ICC 4958 (*C. arietinum*)×PI 489777 (*C. reticulatum*). A BAC-library comprising 55,680 clones was constructed and 46,270 BESs were generated. Mining of these BESs provided 6,845 SSRs, and primer pairs were designed for 1,344 SSRs. In parallel, DArT arrays with ca. 15,000 clones were developed, and 5,397 clones were found polymorphic among 94 genotypes tested. Screening of newly developed BES-SSR markers and DArT arrays on the parental genotypes of the RIL mapping population showed polymorphism with 253 BES-SSR markers and 675 DArT markers. Segregation data obtained for these polymorphic markers and 494 markers data compiled from published reports or collaborators were used for constructing the genetic map. As a result, a comprehensive genetic map comprising 1,291 markers on eight linkage groups (LGs) spanning a total of 845.56 cM distance was developed (http://cmap.icrisat.ac.in/cmap/sm/cp/thudi/). The number of markers per linkage group ranged from 68 (LG 8) to 218 (LG 3) with an average inter-marker distance of 0.65 cM. While the developed resource of molecular markers will be useful for genetic diversity, genetic mapping and molecular breeding applications, the comprehensive genetic map with integrated BES-SSR markers will facilitate its anchoring to the physical map (under construction) to accelerate map-based cloning of genes in chickpea and comparative genome evolution studies in legumes.

## Introduction

Chickpea (*Cicer arietinum* L.) is a self-pollinated, diploid (2*n* = 2*x* = 16), grain legume crop with a genome size of 740 Mb [1]. It is the third most important legume crop of the world and the first most important pulse crop of India (http://www.icrisat.org/crop-chickpea.htm). The *kabuli* types are generally grown in the Mediterranean region including Southern Europe, Western Asia and Northern Africa and the *desi* types are grown mainly in Ethiopia and Indian subcontinent. It is cultivated mostly on low-input and residual moisture from monsoon rains on the Indian subcontinent and semi-arid regions of Sub-Saharan Africa. Chickpeas are high in protein (23%), dietary fiber, carbohydrates (64% of total carbohydrates), and minerals like calcium, magnesium, potassium, phosphorus, iron, zinc and manganese, hence it is considered a neutraceutical crop. Besides terminal drought, *Helicoverpa armigera* (pod borer) is the most devastating pest of chickpea, amounting to annual yield losses to the tune of US$ 400 million per annum in India, and over US$ 2 billion in the semi-arid tropics [2]. Hence, despite the growing demands and high yield potential, chickpea yields are stable and productivity has remained almost stagnant at unacceptably low levels [3], [4].

In spite of tireless efforts of the chickpea breeding community at a global scale, not much progress has been made to overcome these production obstacles. Nevertheless, recent advances in crop genomics offer a great potential for improving crop productivity by deploying marker-assisted selection (MAS) for production constraints in chickpea breeding. Simple sequence repeats (SSRs) or sequence tagged microsatellites (STMS) markers have proven as molecular markers of choice for plant genetics and breeding [5]. In case of chickpea, a few hundred SSR markers were isolated from genomic DNA libraries [6]–[8] or mined from expressed sequence tags (ESTs) [9], [10], and some of them were integrated into genetic maps of chickpea [7], [8], [10]. Similarly, a set of 233 SSR markers were developed after screening a bacterial artificial chromosome (BAC)-library with synthetic oligonucleotides complementary to SSRs [11]. However, only 52 SSR markers were integrated into genetic map [8]. Another method of SSR marker development is the sequencing of BAC-end sequences (BESs), and the resulting SSR markers are referred as BAC-end derived SSR (BES-SSR) markers [12], [13]. Mapping of BES-SSR markers facilitates alignment of genetic and physical maps for applications in map-based cloning and genome sequencing [14], [15].

Diversity arrays technology (DArT), developed by Jaccoud et al. [16], is another approach for screening a large number of marker loci in parallel. DArT markers have been employed for developing high-density genetic maps and assessing genetic diversity at a large scale in several crops, e.g. barley [17], wheat [18], pearl millet [19], to name some. Among legumes, so far DArT markers have only been mapped for pigeonpea [20].

In addition to SSR and DArT marker systems, single nucleotide polymorphism (SNP) markers, because of their higher abundance and amenability for high-throughput approaches are becoming popular as well in crop genetics and breeding [21]. By using allele-specific sequencing for candidate genes and mining the ESTs derived from several genotypes, SNPs have been identified in chickpea [8], [10], [22]. Some of these SNPs have been integrated into genetic maps of chickpea [10].

By using different marker systems, high-density genetic maps have been developed for several crop species including legumes like soybean [23], cowpea [24] and common bean [25]. However, this has not been the case for chickpea, mainly because of the narrow genetic basis of the cultivated gene pool of chickpea. Therefore, the chickpea community has used *C. reticulatum*, a closely related wild species, to develop an inter-specific mapping population for genetic mapping of a maximum number of marker loci. The recombinant inbred line (RIL) mapping population, namely *C. arietinum* (ICC 4958)×*C. reticulatum* (PI 489777), has been extensively used and considered as the reference mapping population for genome mapping [7], [8], [10], [26]. Even based on this mapping population, the most advanced genetic map reported so far, provides the order of maximally 521 markers including SSR and SNP marker loci [8]. Nevertheless, Millán et al. [26] has developed a consensus map based on five inter-specific maps and integrated 555 marker loci including 251 random amplified polymorphic DNAs (RAPDs), 149 STMSs, 47 amplified fragment length polymorphisms (AFLPs), 33 cross-genome markers, 28 gene-specific markers, 10 isozyme markers, 10 inter-simple sequence repeats (ISSRs) and 7 resistance gene analogue (RGA) loci.

With an objective of developing a high-density genetic map based on a single mapping population with maximum genome coverage and precise marker order, the present study reports: (i) construction of a new bacterial artificial chromosome (BAC) library and generation of BAC-end sequences (BESs), (ii) development of novel BES-SSR markers, (iii) development of DArT arrays, and (iv) construction of a dense genetic map based on the BES-SSR and DArT markers (developed in this study) and legacy markers. Genetic mapping data from this study as well as their comparison with two other maps [8], [26] are available in the CMap database at http://cmap.icrisat.ac.in/cmap/sm/cp/thudi/.

## Results

### Construction of BAC-library and generation of BAC-end sequences

The bacterial artificial chromosome (BAC) library (CAA1Ba) was developed from chickpea accession ICC 4958. The library consisted of 55,680 clones, with most inserts ranging from 100 to 130 kbp. A set of 25,000 BAC clones, randomly selected, were sequenced from both ends. Terminal vector sequences were then trimmed and BESs shorter than 100 bp were discarded. As a result, a total of 46,270 high quality BESs were generated. These sequence data are available in the form of genome survey sequences (GSS) at National Center for Biotechnology Information (NCBI) with GenBank accession numbers EI846478.1 to GS878115.1 and GenBank gi numbers 14645554 to 270242271.

### Identification and distribution of BES-SSRs

**Figure 1:**
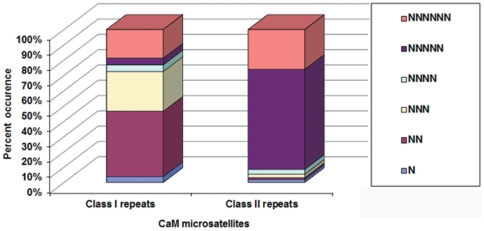
Distribution of Class I and Class II repeats in newly isolated chickpea microsatellites. Class I microsatellites contain >20 nucleotides, Class II repeats perfect SSRs with >12 but <20 nucleotides. Among Class I repeats, tri-nucleotide repeats were most abundant, followed by di-nucleotide repeats, while in Class II repeats, penta-nucleotide repeats were most prevalent, followed by hexa-repeats. N, mono-nucleotide repeats; NN, di-nucleotide repeats; NNN, tri-nucleotide repeats; NNNN, tetra-nucleotide repeats; NNNNN, penta-nucleotide repeats, NNNNNN, hexa-nucleotide repeats.

**Figure 2:**
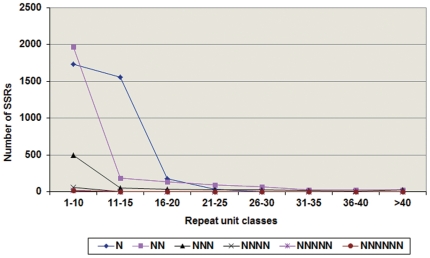
Distribution of microsatellites with varying repeat units in BAC-end sequences. N: mono-nucleotide repeats; NN: di-nucleotide repeats; NNN: tri-nucleotide repeats; NNNN: tetra-nucleotide repeats; NNNNN: penta-nucleotide repeats and NNNNNN: hexa-nucleotide repeats.

With an aim of increasing the molecular marker repertoire for chickpea, 46,270 BESs representing 33.22 Mbp of the genome were surveyed for the presence of SSRs by means of the MIcroSAtellite (*MISA*) search module ([27], http://pgrc.ipk-gatersleben.de/misa/). In total 6,845 SSRs were identified in 5,123 BESs, scanning one SSR per every 4.85 kb. The SSRs were either perfect (i.e., containing a single repeat motif such as ‘ATA’) or compound SSRs (i.e., composed of two or more SSRs separated by ≤100 bp). About 1,245 BESs contained more than one SSR motif, while 913 SSRs identified were in compound form. Perfect SSRs were further subdivided according to the length of SSR tracts [28], [29]: Class I SSRs (≥20 nucleotides in length) and Class II SSRs (≥10 but <20 nucleotides in length). Among Class I repeats, di-nucleotide repeats (42.7%) were most abundant, followed by tri-nucleotide repeats (26%), while Class II repeats consisted mostly of penta-nucleotides (65.30%), followed by hexa-nucleotide repeats (26.10%; Figure 1). Among the SSR repeats, mono-nucleotide (51.35% of total) and di-nucleotide repeats (37.03% of total) were dominating. Excluding mono-nucleotide repeats, which were almost exclusively poly-A motifs, A/T-rich repeats accounted for 49.84% of all SSRs. The frequency of AT-rich repeats increased as motif length increased, from a low of 71.18% in di-nucleotide repeats to a high of 94.75% in hexa-nucleotide repeats. Majority of the SSR motifs occurred in the range of <10 to 20 repeat units category (Figure 2).

### Development of novel genetic markers

Out of 6,845 SSRs identified in 5,123 BESs, the primer pairs were designed for 2,189 non-redundant BES-SSRs (Table S1). The markers based on these primer sequences have been referred as *C*icer *a*rietinum *M*icrosatellite (CaM) markers. However, based on criteria mentioned in our earlier study [8] for getting higher proportion of polymorphic markers, only 1,344 primer pairs were synthesized and tested for amplification and polymorphism potential. Primer sequence information, repeat motifs, amplicon sizes, and polymorphism features for all 1,344 primers are provided in Table S1. In addition, primer sequence information is also provided in Table S1 for 845 primers pairs that were not characterized in the present study so that the chickpea community can utilize the developed resource.

**Figure 3:**
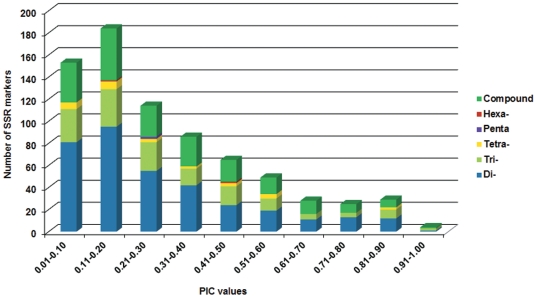
Number of BES-SSR markers in different PIC value classes. The number of di-, tri-, tetra-, penta-, hexa- nucleotide repeats and compound SSRs in different PIC value classes are in blue, light blue, yellow, purple, dark red and green respectively.

Of 1,344 primer pairs tested on the two genotypes ICC 4958 and ICC 1882, scorable amplification was observed with 1,063 primer pairs. Furthermore, 737 (69.33%) primer pairs or markers showed polymorphism on a panel of 48 genotypes including 33 genotypes from cultivated species (*C. arietinum*) and 15 genotypes from eight wild species including *C. echinospermum, C. bijugum, C. cuneatum, C. judaicum, C. microphyllum, C. pinnatifidum, C. reticulatum* and *C. yamashitae* (Table S2). In terms of polymorphism detection, markers derived from hexa-nucleotide repeats were highly polymorphic followed by tetra-, penta-, tri- and di-nucleotide repeats. In brief, 69.33% (737) markers were polymorphic and detected a total of 3,144 alleles ranging from 2–25 with an average of 4.26 alleles per marker locus. The PIC value of these polymorphic markers ranged from 0.04 to 0.94 with an average of 0.30. Of 737 polymorphic markers, 602 markers had a PIC value of ≤0.50 and a set of 86 (11.66%) highly informative SSR markers with PIC values >0.60 was identified (Figure 3).

Among 737 polymorphic markers, 517 were polymorphic in 15 genotypes of eight wild species and 329 markers were polymorphic across 33 genotypes of the cultivated species. As expected, a higher level of polymorphism was detected in inter-specific crosses as compared to intra-specific crosses. For instance, 126 – 253 markers showed polymorphism between parents of inter-specific mapping populations, while 99 – 171 markers displayed polymorphism between parents of intra-specific mapping populations (Table 1).

**Table 1:** Polymorphism survey of novel SSR and DArT markers between parental genotype combinations of different intra- and inter-specific mapping populations.

	BES-SSR markers (total 1,063 used)			DArT markers (total 15,360 clones used)		
Crosses	Markers amplified	Number of polymorphic markers	Polymorphism (%)	Number of DArT clones giving signals	Number of polymorphic markers	Polymorphism (%)
Intra-specific (*C. arietinum*×*C. arietinum*)						
ICC 4958×ICC 1882	931	100	10.74	5,285	496	9.38
ICC 283×ICC 8261	864	159	18.40	5,308	327	6.16
ICCV 2×JG 11	909	99	10.89	5,368	35	0.65
ICCV 2×JG 62	882	171	19.39	5,380	36	0.66
ICC 506EB×Vijay	949	117	12.33	5,303	99	1.86
ICC 6263×ICC 1431	913	128	14.02	5,352	447	8.35
Inter-specific (*C. arietinum*×*C. reticulatum*)						
ICC 4958×PI 489777	990	253	25.55	5,262	675	12.82
ICC 3137×IG 72953	931	129	13.86	5,299	680	12.83
ICC 3137×IG 72933	863	126	14.60	5,266	210	3.98
ICC 8261×ICC 17160	848	229	27	5,248	845	16.10
ICCV 2×ICC 17160	823	248	30.13	5,262	906	17.21

### Development of DArT markers

A DArT array with 15,360 DArT clones was developed from a *Pst*I*/Taq*I representation generated from a mixture of DNA of 94 diverse chickpea genotypes as well as some other chickpea genotypes of Australian origin. After scanning the developed DArT arrays on the set of 94 genotypes, a total of 5,397 DArT markers exhibited polymorphism. The number of polymorphic markers among parents of different intra- and inter-specific mapping populations ranged from 35 to 496 and 210 to 906, respectively (Table 1). Only 675 DArT markers showed polymorphism between the parental genotypes of the inter-specific mapping population (ICC 4958×PI 489777).

**Figure 4:**
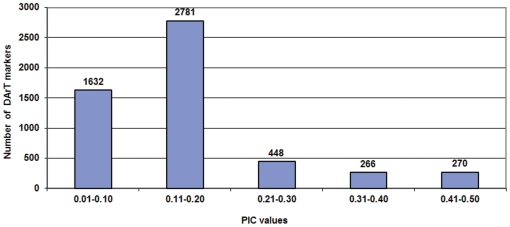
Number of DArT markers in different PIC value classes. Polymorphic markers have been grouped into five classes of PIC values namely 0.01– 0.10, 0.11–0.20, 0.21– 0.30, 0.31– 0.40 and 0.41–0.50.

The PIC values for DArT markers were relatively low, with only 11.72% of DArTs having PIC values of 0.30–0.50, whereas 81.7% DArTs exhibited PIC values of <0.20 (Table S3). The average mean PIC value was 0.13. Further, when the quality of the DArT markers was analyzed against their performance, which is determined by call rate and PIC values, 34.64% of the polymorphic DArT markers (*n* = 1,870) were in the 80–100% quality category with an average PIC value of 0.18 and a call rate of 99.36%, respectively (Table S3). The average PIC value decreased with the average quality value. The PIC values for 108 markers possessing marker quality of <50% ranged from 0.02 – 0.14. Of 1,870 markers with a quality of more than 80%, only 328 markers had a PIC value of >0.30 (Table S3). Out of 5,397 polymorphic DArT markers in the germplasm analyzed, there are 266 and 270 markers in the PIC value range of 0.30–0.40 and 0.40–0.50, respectively (Figure 4).

### Construction of a high-density inter-specific map

**Figure 5:**
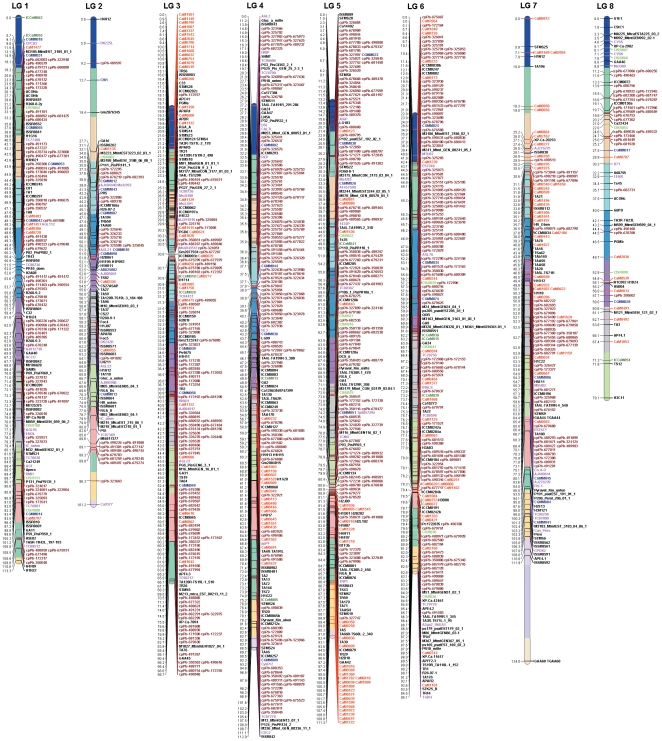
Interspecific reference genetic map with 1,291 loci, spanning 845.56 cM. The map distance is indicated on the left and the marker names on the right side of each linkage group. Each linkage group is divided into 10 cM bins. Marker series are colour coded: CaM (red), DArT (brown), ICCeM (green), CISR (light green), COS-SNP (pink), CAPS (blue) and legacy markers (black).

With an objective to construct a high-density genetic map of chickpea, newly developed BES-SSR markers and DArT arrays were screened on the parental genotypes, i.e. ICC 4958 and PI 489777 of the reference mapping population. As a result, 253 BES-SSR and 675 DArT markers were polymorphic between the parental genotypes. Subsequently, segregation data were obtained for all 928 polymorphic markers on 131 RILs of the mapping population. In addition, genotyping data were collected for 192 genic molecular markers (GMMs) including 83 conserved orthologous sequences (COS)-based SNPs (COS-SNPs), 54 cleaved amplified polymorphic sequences (CAPS), 35 conserved intron spanning region (CISR) and 20 EST-derived SSR (EST-SSR, with the name ICCeM) marker loci published in Gujaria et al. [10] and 494 first generation DNA markers that have been used in construction of genetic maps in several studies [8], [10], [30], referred as legacy markers. In summary, genotyping data obtained for all 1,614 markers were compiled and used for developing the genetic map. In the first instance, all the markers showing good segregation were used for the construction of the genetic map. Subsequently, with an objective of not losing the genetic information of other published markers, the markers showing segregation distortion were also tried to integrate into the map (Table S4). Finally, a total of 1,291 (79.99%) marker loci were mapped onto eight linkage groups (LGs) spanning a distance of 845.56 cM (Table 2; Figure 5; http://cmap.icrisat.ac.in/cgi-bin/cmap_public/viewer?data_source=CMAP_PUBLICsaved_link_id=5). The linkage groups have been numbered LG 1 - LG 8 following the nomenclature style of our earlier study [8].

**Table 2:** Distribution of different type of markers on eight chickpea linkage groups (LGs).

	NovelSSR markers (CaM)	Genic molecular markers (GMMs)				DArT markers	Legacy markers	Total
Marker series		EST-SSR	CISR	CAPS	COS-SNP			
**Markers used**	253	20	35	54	83	675	494	1,614
**Total markers mapped**	157	11	18	35	81	621	368	1,291
**Percent mapped**	62.06	55	51.43	64.81	97.59	92	74.49	79.99
**Markers unlinked**	96	9	17	19	2	54	126	323
**Percent unlinked**	37.94	45	48.57	35.19	2.41	8	25.51	20.01
**Markers mapped on different linkage groups (LGs)**								
LG 1	6	2	3	4	16	77	48	156
LG 2	3	-	1	8	8	26	52	98
LG 3	34	2	1	3	10	91	52	193
LG 4	17	2	-	7	11	122	53	212
LG 5	32	1	4	3	8	114	56	218
LG 6	25	3	5	4	8	119	45	209
LG 7	33	-	-	4	13	47	40	137
LG 8	7	1	4	2	7	25	22	68
**Total**	**157**	**11**	**18**	**35**	**81**	**621**	**368**	**1,291**

In summary, the developed genetic map in this study comprises 157 novel SSR loci, 621 novel DArT loci, 145 GMM, and 368 legacy marker loci (Table 2). The number of markers per linkage group varied from 68 (LG 8) to 218 (LG 5). The Figure 5 shows distribution of all the marker loci, as mentioned above, on 8 linkage groups (LGs). The length of individual linkage groups ranged from 79.06 (LG 8) to 133.97 cM (LG 7). LG 5 had the highest number of marker loci (218) while the highest map length was recorded for LG 7 (133.97 cM). On the other hand, LG 8 exhibited the lowest number of mapped markers (68) as well as the shortest map distance (79.06 cM). On average, one marker is present for every 0.65 cM per each linkage group.

Of 621 DArT loci mapped, 355 (57.2%) loci were mapped on only three linkage groups (LG 4, LG 5 and LG 6). Maximum number of novel CaM marker loci was mapped on LG 3 (34) followed by LG 7 (33) and LG 5 (32). None of the EST-SSR (ICCeM) markers was mapped onto LG 2 and LG 7, and similarly none of the CISR markers was mapped to LG 4 and LG 7. For making the map more informative for selecting the markers for future genetic mapping and diversity analysis studies in chickpea, each LG was divided into 10 cM long bins (Figure 5). The PIC value and number of alleles, wherever possible, were calculated for all the mapped markers. The average PIC value of the mapped SSR markers on individual LGs varied from 0.29 (LG 6) to 0.51 (LG 1), while the average number of alleles ranged from 4 (LG 2) to 6.33 (LG 8) (Table S5). The information on PIC values and number of alleles associated with the SSR markers in different bins will help selection of highly informative SSR markers from each bin in a systematic way that will represent the genome as well as enhance the probability of displaying high polymorphism in the germplasm to be analyzed.

Uneven distribution was observed for the mapped markers across all linkage groups (Figure 5). A total of 59 major clusters (≥5 loci/cM) were identified on all eight linkage groups (Table 3). The largest cluster included 25 loci within 1 cM interval on LG 3. Furthermore, at least one cluster of DArT loci was found on each linkage group in the current map. A maximum of 13 clusters comprising 97 marker loci was observed on LG 5. Uneven distribution of markers was also evident from the occurrence of gaps in different linkage groups. A total of 16 minor gaps (5 –10 cM) between adjacent markers were spread across seven linkage groups (Table 3), except for LG 3. A large gap between adjacent markers (>20 cM) was observed on LG 7, and a gap >10 cM on LG 4. Nevertheless, 16 gaps between 5 and 10 cM on all LGs exist, except for LG 5 (Figure 5; and Table 3).

**Table 3:** Features of the inter-specific reference genetic map.

Linkage group (LG)	Number of markers	Length (cM)	Density (markers/cM)	Number of intervals	Number of gaps (>5 cM)	Number of clusters	Genetic mapping position and number of markers (in parenthesis) in clusters observed
LG 1	156	113.32	0.73	121	2	9	18 cM (6), 44 cM (8), 47 cM (5), 8 cM (7), 49 cM (6), 54 cM (6), 2 cM (8), 72 cM (7), 91 cM (6)
LG 2	98	101.19	1.03	84	4	3	50 cM (5), 52 cM (5), 89 cM (8)
LG 3	193	98.66	0.51	153	1	8	21 cM (7), 30 cM (12), 31 cM (25), 32 cM (6), 36 cM (5), 42 cM (7), 60 cM (7), 79 cM (5)
LG 4	213	112.91	0.53	160	1	10	15 cM (6), 34 cM (8), 35 cM (12), 36 cM (15), 38 cM (9), 39 cM (6), 40 cM (5), 59 cM (9), 61 cM (11), 98 cM (9)
LG 5	218	111.29	0.51	160		14	5 cM (5), 6 cM (9), 11 cM (8), 26 cM (5), 52 cM (6), 56 cM (9), 63 cM (9), 74 cM (9), 79 cM (8), 80 cM (6), 81 cM (8), 82 cM (10), 87 cM (5), 100 cM (6)
LG 6	208	95.12	0.46	149	2	11	10 cM (9), 22 cM (5), 38 cM (5), 45 cM (16), 46 cM (6), 47 cM (6), 67 cM (6) 78 cM (24), 79 cM (10), 82 cM (6), 87 cM (5)
LG 7	137	133.97	0.98	109	5	4	31 cM (9), 52 cM (6), 53 cM (17), 68 cM (6)
LG 8	68	79.06	1.16	59	1	1	20 cM (6)
**Total**	**1,291**	**845.56**	**-**	**995**	**16**	**60**	
**Average**	**161.38**	**105.70**	**0.74**	**124.38**	**2**	**7.5**	

To assess the congruency of marker order and map position, the present comprehensive genetic map was compared with four earlier genetic maps [7], [8], [26], [31]. On comparison, the linkage group position of different markers remained conserved in case of LG 2, LG 3, LG 4, LG 5 and LG 8 with Nayak et al. [8]. However, markers on LG 1, LG 6 and LG 7 of the present map exhibited some discrepancies in their position. For instance, of 218 markers mapped on LG 5, in the current study, 35 were present on LG 5 and 10 markers on LG 2 of Nayak et al. [8]. LG 1, LG 2 and LG 4b of the consensus map of Millán et al. [26] based on narrow (intra-specific) crosses correspond to LG 1, LG 2 and LG 4 of the current map (Table 4). Similarly, LG 1, LG 3, LG 4, LG 5 and LG 6 of the consensus map based on wide (inter-specific) crosses correspond to LG 1, LG 3, LG 4, LG 5 and LG 6 of the present map. The linkage groups LG 4 and LG 11 of Winter et al. [7] correspond to LG 4 of present map. The linkage group wise correspondence among current map and the maps developed by Winter et al. [7] and Millán et al. [26] have been shown via CMap (http://cmap.icrisat.ac.in/cmap/sm/cp/thudi/).

**Table 4:** Comparison of the linkage groups (LGs) of the present reference genetic map with some key genetic maps.

Map developed in this study	Winter et al. [7]	Radhika et al. [31]	Nayak et al. [8]	Millán et al. [26]	
				Narrow crosses	Wide crosses
**LG 1**	LG 1	LG 2	LG 1, LG 3	LG 1	LG 1
**LG 2**	LG 2	LG 2, LG 3	LG 2	LG 2	LG 1, LG 2
**LG 3**	LG 3	LG 1	LG 3	-	LG 3
**LG 4**	LG 4, LG 11	LG 1, LG 2	LG 4	LG 4b	LG 4
**LG 5**	LG 7	LG 1	LG 5	-	LG 5
**LG 6**	LG 6	LG 1, LG 4	LG 2, LG 6	-	LG 6
**LG 7**	LG 5, LG 9, LG 15	LG 5	LG 5, LG 7	-	-
**LG 8**	LG 8	LG 3, LG 6	LG 8	-	-

## Discussion

### Novel SSR markers from BESs

A new 10X BAC library and 46,270 BESs have been generated for the reference genotype ICC 4958 in the present study. Although BAC libraries have been targeted for isolation of SSRs in chickpea earlier [11], [32], this is the first time that SSR markers have been developed after mining the BESs. This study adds a new set of 1,063 BES-SSR markers of which 737 markers showed polymorphism in the set of 48 tested genotypes, of which 58 markers with a PIC value of >0.70 were highly informative. In terms of mapping newly developed BES-SSR markers to the genetic map, success was obtained only in the case of 157 (11.68%) markers. This reduction in number of markers from designing the primer pairs to mapping is termed as “SSR attritions” [33]. Higher attrition rates have also been reported earlier in the case of BES-SSR markers e.g. rye [34]. Nevertheless, one of the most important advantage of the developed BES-SSR markers over genomic or EST-SSR markers is that they serve as anchor points between genetic and physical maps [13], [25], [35]. Screening of these markers on a set of parental genotypes of 11 mapping populations provided 99 to 253 polymorphic markers in different intra- and inter-specific mapping populations (Table 1). These markers can be used for map construction and trait mapping in the respective populations.

In the total set of 6,845 SSRs identified in 5,123 BESs, the Class I SSRs (≥20 nucleotides in length) include a higher proportion of di-nucleotide repeats (42.7%), followed by tri-nucleotide repeats (26%), while Class II SSRs were mostly derived from penta-nucleotides (65.3%), and followed by hexa-nucleotides (26.1%). Availability of information on this aspect of SSRs is important for the selection of potential polymorphic SSR markers. In case of ICCM markers, the average PIC value of Class I SSRs was higher (0.38) than that of Class II SSRs (PIC = 0.22), thus demonstrating the potential of Class I SSRs over Class II SSRs [8]. Similarly, in the case of CaM markers, average PIC value of Class I SSRs was higher (0.21) compared to Class II SSRs (0.11). The majority of Class I SSRs contains tri-nucleotide repeats, indicating the importance of tri-nucleotide repeat motifs over others.

SSR frequency in the present study was found to be one SSR in every 4.85 kb. The frequency and distribution of SSRs, however, depends on various factors such as size of sequence dataset, tools and criteria used [36]. As a result, in the same species, a varied level of frequency of SSRs has been reported in different studies [36]. Similar is the case of chickpea where SSR frequencies have been reported as 1/707 bp in coding regions [9], 1/1.3 kb in transcriptome assembly [37] and 1/4.85 kb in BESs in the present study.

In general, tri-nucleotide repeats were considered the most polymorphic sites [36]. In addition to tri-nucleotide repeats, compound SSRs constituted the majority of polymorphic markers during the present study. Contrary to majority of the other plant species where di-nucleotide repeats showed high polymorphism [27], [38], hexa-nucleotide repeats were highly polymorphic in the present study. Similar results have been reported in the case of pigeonpea [39] and common bean [40]. PIC values of compound SSRs (average PIC values of ICCM = 0.29 [8] and CaM = 0.27) were comparable to those of tri-nucleotide repeats. This can be attributed to the fact that the markers with compound SSRs have more than one SSR motif, which increases their chances to be polymorphic [8]. The present study demonstrated a positive correlation between number of alleles and PIC values. For instance, CaM0713 produced the highest number of alleles (25) with highest PIC values (0.94) followed by CaM0836 with 21 alleles and PIC value of 0.93.

### DArT marker system for chickpea

DArT markers are typically developed from a representation that is generated from a pool of DNA samples from a number of accessions, cultivars or breeding lines which as a group represent the genetic diversity within a species [16]. In the current study, high-density DArT arrays comprising of 15,360 clones were generated from genomic representations of 94 diverse genotypes (used as parents of mapping populations), genotypes from the reference set and wild genotypes exploited for introgression studies. A total of 5,397 (35.13%) markers were found polymorphic on the panel of 94 genotypes. Thus it is very evident that, compared to other marker technologies, DArT markers can be developed and typed quickly and cheaply [20]. The PIC values of DArT markers for chickpea germplasm are comparable to other germplasms such as sorghum [41] and cassava [42]. Ten percent of DArT markers had a PIC value in the range of 0.30 to 0.50, and these markers, therefore, may be considered useful or informative.

### The most comprehensive genetic map of the chickpea genome

Despite the availability of a few hundred SSR markers in chickpea, putting them on the genetic map has been a challenging task due to the low level of polymorphism in cultivated chickpea germplasm [43]. MAS is most effective when markers are tightly linked to the gene of interest so that the probability of crossing-over between the gene and markers decreases. Moreover, map-based cloning requires very fine resolution mapping in the target interval, since the highest marker density can shorten chromosome walking. Hence, an inter-specific mapping population derived from ICC 4958 (*C. arietinum*) and PI 489777 (*C. reticulatum*) was used to integrate the novel markers developed in this study together with earlier published or some unpublished markers. This mapping population has been widely used by the chickpea community to incorporate several hundred microsatellite [7], [8] and gene based markers [10], [44]. The diverse genetic background of the parents showed higher degree of polymorphism not only at the genetic level but also at phenotypic levels such as resistance to *Fusarium* wilt [7] and *Ascochyta* blight [45], thus, facilitating trait mapping. Consequently, this population could generally be considered as the “international reference” mapping population [8].

The integrated genetic map developed in this study comprises 157 novel SSR (CaM loci), 621 novel DArT marker loci, 145 GMM (81 COS-SNP, 35 CAPS, 18 CISR and 11 ICCeM) loci [10] and 368 legacy marker loci [7], [8], [11], [46]. The current map with 1,291 loci is the most comprehensive genetic map ever reported for chickpea. Although the direct comparison of this map with the other published maps is not possible as different studies use different mapping programmes and criteria, the smaller map distance (845.56 cM) of the current map as compared to other published maps so far on this population indicates that this map is probably the densest genetic map for chickpea. Higher marker density can be attributed to: (i) the integration of large number of markers including both developed in this study and from other studies, and (ii) the use of JoinMap v 4 for calculating the map distance. In general, the maps constructed with JoinMap are shorter than those generated with a multi-locus likelihood package such as MAPMAKER [47]. The multi-locus likelihood method used by MAPMAKER assumes an absence of crossover interference, and, JoinMap allows interference and correctly produces shorter maps, even though both programs use the Kosambi mapping function [48]. The marker density of each individual linkage group ranged from 1 marker/0.46 cM (LG 6) to 1 marker/1.16 cM (LG 8). No correlation was found between the number of mapped markers and the length of linkage groups. For instance, LG 3 spans a distance of 98.66 cM with 193 markers, but LG 2 with only 98 loci spans a distance of 101.19 cM (Table 3). Similar observations were recorded in earlier chickpea mapping studies [7], [8], [26].

It is important to mention that the developed map has 94 marker loci that showed segregation distortion (p≤0.05). These markers have been retained in the map intentionally so that genetic information associated with such markers mapped in past (33 legacy markers and 8 GMM markers) or future (11 CaM and 42 DArT markers) may not be lost (Table S4). The legacy markers showed segregation distortion in earlier genetic mapping studies [7], [8], [26] and DArT markers have also shown segregation distortion in several species like triticale [49], wheat [50]. Comparison of this map with other key genetic maps [7], [8], [26], [31] that contained majority of legacy markers showed a good congruency in terms of both marker orders as well as nomenclature of linkage groups was observed. These observations reconfirm the quality of the map and rule out the possibility of being concerned with the markers showing segregation distortion on the genetic map. As this map is the densest genetic map and includes the majority of the mapped loci available on the genetic map, this map could be considered and used as the reference genetic map of chickpea for developing and comparing new genetic maps in future.

The utility of our dense map could be demonstrated by fine mapping of locus CS27, the resistance locus for *Fusarium* wilt race 1 (*Foc1*), that was mapped onto LG 2 by Winter et al. [7] and the same LG of the present map. This locus is also linked to *Fusarium* race 4 (*Foc4*) and 5 (*Foc5*) at distances of 0.57 and 2.44 cM, respectively, on the current map. However, race 4 and 5 were 3.7 and 21.5 cM, respectively, away from locus CS27 in the map of Winter et al. [7]. Clustering of resistance genes for different races of pathogens and also different pathogens has been demonstrated in different crop plants including legumes [46]. The SSR markers flanking CS27, *Foc4*, *Foc5*, for instance TA37, H1J07 and CaM0955 and other markers on either side of these loci can be employed in marker-assisted breeding programs. Of five resistance gene analogs (RGAs; RGA-D, RGA-Ds, RGA-A, RGA-C, RGA-B and RGA-G) mapped onto four linkage groups of chickpea [46], namely LG 2, 3, 5 and 6, only four RGAs (RGA-D, RGA-Ds, RGA-A, RGA-C, RGA-B) could be mapped onto three respective linkage groups (LG 2, LG 3 and LG 5).

With an objective of enhancing the utility of the reference genetic map for genetics research and breeding applications, the reference genetic map developed here has been divided into bins of 10 cM length. For several marker loci from these bins, we also have the information on PIC values or number of alleles. Information on distribution of marker loci into different bins along with the polymorphism features is an added-value. This will help geneticists and breeders to select an informative set of markers in appropriate numbers that represent the genome as well as display a high degree of polymorphism for developing new genetic maps, trait mapping and diversity analysis.

### Uneven distribution of recombination in the chickpea genome

The present map indicates that recombination in chickpea, like some other plant species, is unevenly distributed with “hot-spots” and “cold-spots” across chromosomes. Clustering around centromeres is a well-known phenomenon with all types of markers, resulting from centromeric recombination suppression [51]. A set of 11 DArT markers were clustered near the centromeric region of LG 1. A remarkable clustering of DArT and BES-SSR markers was found in telomeric regions of LG 3 and LG 5. Although such clustering of markers was not reported in earlier mapping studies in chickpea, a stronger tendency of DArT markers towards clustering, as compared to SSR markers, in particular in gene-rich telomeric regions was shown in some crop species like wheat [52] and barley [53]. Markers sometimes tend to cluster, either as a consequence of an uneven distribution of recombination events along chromosomes, or because markers preferentially survey DNA polymorphism that is unevenly distributed along chromosomes [54], [55]. For instance, clustering of *Pst*I-based DArT markers may reflect the abundance of *Pst*I restriction sites in hypomethylated telomeric chromosome regions [56].

In summary, this study reports the development of a set of 1,063 novel BES-SSR markers, of which 737 were polymorphic in the surveyed germplasm, and 157 could be integrated into the genetic map. Similarly, a DArT array with 15,360 clones was developed of which 5,397 were polymorphic in the surveyed germplasm, and 621 DArT loci were mapped. Using the above mentioned BES-SSR and DArT marker loci together with other marker datasets, a comprehensive genetic map with 1,291 marker loci has been developed. It is anticipated that the new markers and the dense genetic map will be useful for genetic analysis and breeding of chickpea and for the comparative study of genome evolution in legumes.

## Materials and Methods

### Plant material and DNA extraction

The reference chickpea genotype ICC 4958 was used for the construction of the BAC library. The developed set of BES-SSR markers were screened on ICC 4958 and ICC 1882, the parental genotypes of an intra-specific mapping population, for the amplification of SSR loci. Subsequently, a set of forty eight chickpea genotypes listed 1–48 in Table S2, were used for identification and characterizing of an informative set of the BES-SSR markers.

For the development of DArT arrays, a set of 94 genotypes (19-112 genotypes listed in Table S2) including parental genotypes of several mapping populations, diverse accessions from the reference set [57] and 19 accessions of wild *Cicer* species were used.

An F_10_ population comprising 131 recombinant inbred lines (RILs), derived from the inter-specific cross of ICC 4958 (*Cicer arietinum*) and PI 489777 (*C. reticulatum*), was used for screening and genotyping with the newly developed set of BES-SSR and DArT markers in this study and with the H-series markers [11].

DNA was extracted from the two weeks old seedlings of above mentioned genotypes using a high-throughput mini-DNA extraction as mentioned in Cuc et al. [58].

### Construction of BAC library and generation of BAC-end sequences

The accession ICC 4958 was grown under greenhouse conditions for 6 weeks and transferred to continuous darkness for 2 days prior to use. Nuclei were isolated and embedded in low melting agarose, restriction digested with *Hin*dIII and size selected by two rounds of pulsed field gel electrophoresis (PFGE). Large size DNA fragments were ligated into vector pCCBAC1H and transformed into Epicenter's *E. coli* EPI300-T1R cells by electroporation.

A set of 25,000 BAC clones from the above library was prepared for end-sequencing at the J. Craig Venter Institute (JCVI), USA. Base calling and sequence trimming were performed as described in Bohra et al. [59].

### Mining of SSRs in BESs and primer design

BESs were used for mining the SSRs with Perl based *MI*cro*SA*tellite (*MISA*) ([27], http://pgrc.ipk-gatersleben.de/misa/) search module which is capable of identifying perfect as well as compound SSRs. All BESs with a minimum size of 100 bp were arranged in a single FASTA format text file, and this file was used as an input for *MISA*. True and compound SSRs were classified through criteria defined by Nayak et al. [8].

In general, one SSR-containing BES was selected from each cluster for the design of the primer pairs, employing standalone Primer3 (http://frodo.wi.mit.edu/) program using *MISA* generated Primer3 input file [27].

### SSR amplification and analysis

All BES-SSR markers and the H-series SSR markers were used for screening polymorphisms between the parents of the inter-specific mapping population. Subsequently the polymorphic SSR markers were applied to genotype all RILs. PCR amplification conditions and size separation procedures were the same as described in our earlier studies [8], [59].

### Development and genotyping of DArT arrays

A 15,360-clone DArT genomic library (‘diversity array’- forty 384-well plates) was developed from a mixture of DNA samples of 94 chickpea genotypes included in the study (Table S2). Genomic representations for the diversity panel and genotyping were prepared by the complexity reduction method described by Yang et al. [60]. Briefly, ca. 100 ng of DNA were digested with restriction enzymes *Pst*I and *Hae*III (New England Biolabs, USA) and the *Pst*I adapter was simultaneously ligated. One µl of restriction/ligation reaction served as a template in a 50 µl amplification reaction with *Pst*I+0 primer. Adaptor and primer sequences and cycling conditions are given in the earlier study [60]. Arrays were hybridized with fluorescently labeled targets from all genotypes used for the array development [20], [60].

For mapping the DArT markers, genomic representations were generated for all 131 RILs employing the same complexity reduction method (*Pst*I/*Hae*III) mentioned above. After overnight hybridization at 62°C, the slides were washed and scanned with a Tecan LS300 confocal laser scanner (Grödig, Salzburg, Austria). Individual samples were processed identically to samples for marker discovery and with similar marker quality thresholds in DArTsoft analysis [61].

### Polymorphism information content (PIC) value

The PIC values for the SSR and DArT markers were calculated as mentioned in our earlier studies [8], [41].

### Linkage mapping

The genotyping data generated in this study as well as from other published studies [7], [8], [30] and collaborators were used for map construction with JoinMap v 4.0 ([62], www.kyazma.nl/index.php/mc.JoinMap). Prior to map construction, segregation ratios for both alleles (1∶1) were tested for goodness of fit to assess deviations from the expected Mendelian segregation for all markers. Initially, markers showing goodness of fit were used for map construction, but later on markers showing segregation distortion were also attempted to be integrated into the map however always on >LOD 3.0. Linkage groups were determined based on “Independence test LOD score”. Placement of markers into different linkage groups was done with “LOD groupings” and “Create group using the mapping tree” commands. Map calculations were performed with parameters like LOD value ≥2.0, recombination frequency ≤0.40 and a chi-square jump threshold for removal of loci = 5. Addition of a new locus may influence the optimum map order; hence a “Ripple” (enables to identify “the best marker order” by computing goodness-of-fit among three adjacent markers, for each order of the map) was performed after adding each marker into map. Map distances were calculated by the Kosambi mapping function [49], and the third round was set to allow mapping of an optimum number of loci into the genetic map. Mean chi-square contributions or average contributions to the goodness of fit of each locus were also checked to determine the best fitting position for markers in the genetic map. The markers showing negative map distances and large jumps in mean chi-square values were discarded from mapping. The final map was drawn with the help of MapChart v 2.2 [63]. The marker order of the current map was compared with already published maps using CMap v 1.01 (http://cmap.icrisat.ac.in/cmap/sm/cp/thudi/).

## Supporting Information

Table S1 **Details on novel simple sequence repeat (SSR) markers developed after mining bacterial artificial chromosome (BAC)- end sequences.**
(XLSX)

Table S2 **List of 112 chickpea genotypes used for SSR and DArT analysis.**
(XLS)

Table S3 **Features of 5,397 polymorphic DArT loci based on marker analysis in 94 genotypes.**
(XLSX)

Table S4 **Details on mapped markers showing segregation distortion (p<0.05).**
(XLSX)

Table S5 **Bin wise description of the inter-specific reference genetic map of chickpea.**
(XLSX)
